# Gene transduction in mammalian cells using *Bombyx mori* nucleopolyhedrovirus assisted by glycoprotein 64 of *Autographa californica* multiple nucleopolyhedrovirus

**DOI:** 10.1038/srep32283

**Published:** 2016-08-26

**Authors:** Tatsuya Kato, Saki Sugioka, Kohei Itagaki, Enoch Y. Park

**Affiliations:** 1 Laboratory of Biotechnology, Green Chemistry Research Division, Research Institute of Green Science and Technology, Shizuoka University, 836 Ohya Suruga-ku, Shizuoka, 422-8529, Japan; 2 Laboratory of Biotechnology, Department of Applied Biological Chemistry, Faculty of Agriculture, Shizuoka University, 836 Ohya, Suruga-ku, Shizuoka, 422-8529, Japan; 3 Laboratory of Biotechnology, Department of Bioscience, Graduate School of Science and Technology, Shizuoka University, 836 Ohya, Suruga-ku, Shizuoka, 422-8529, Japan

## Abstract

*Autographa californica* multiple nucleopolyhedrovirus (AcMNPV), an alphabaculovirus, has been widely utilized for protein expression in not only insect cells but also mammalian cells. AcMNPV is closely related to *Bombyx mori* nucleopolyhedrovirus (BmNPV), and nucleotide sequences of AcMNPV genes have high similarity with those of BmNPV. However, the transduction of BmNPV into mammalian cells has not been reported. In this study, we constructed a recombinant BmNPV (BmNPVΔbgp/AcGP64/EGFP) whose surface 64 kDa glycoprotein (BmGP64) was substituted with that from AcMNPV (AcGP64). BmNPVΔbgp/AcGP64/EGFP also carried an EGFP gene under the control of the CMV promoter. BmNPVΔbgp/AcGP64/EGFP successfully transduced HEK293T cells. In comparison, a control construct (BmNPVΔbgp/BmGP64/EGFP) which possessed BmGP64 instead of AcGP64 did not express EGFP in HEK293T cells. The transduction efficiency of BmNPVΔbgp/AcGP64/EGFP was lower than that of an AcMNPV based-BacMam GFP transduction control. This result indicates that AcGP64 facilitates BmNPV transduction into HEK293T cells. BmNPV can be prepared easily on a large scale because BmNPV can infect silkworm larvae without any special equipment, even though specific diet is needed for silkworm rearing. BmNPV gene transduction into mammalian cells can potentially be applied easily for gene delivery into mammalian cells.

Baculoviruses belong to the family *Baculoviridae* and are divided into four genera. *Autographa californica* multiple nucleopolyhedrovirus (AcMNPV), which is an alphabaculovirus, one of the four genera of the *Baculoviridae*, has been widely utilized for recombinant protein expression using cultured insect cells^1^. Improvements to AcMNPV have been made to express recombinant proteins efficiently^2^,^3^. Another alphabaculovirus member, *Bombyx mori* nucleopolyhedrovirus (BmNPV), which infects silkworms, has also been utilized for recombinant protein expression with some modifications^4^,^5^.

Baculoviruses can infect invertebrates but not vertebrates. However, it has been reported that AcMNPV can enter various mammalian cells and express recombinant proteins if the coding genes are inserted into its genome under the control of a mammalian or virus-derived promoter^6^,^7^,^8^. These recombinant AcMNPVs are called “BacMam” vectors. Using these vectors, recombinant proteins are expressed in mammalian cells without the replication of recombinant AcMNPV, which minimizes contamination of recombinant AcMNPV in recombinant proteins, unlike what occurs in insect cells^9^.

Glycoprotein 64 (GP64), which resides on the surface (envelope) of AcMNPV, mediates the entry of AcMNPV into mammalian cells through dynein- and clathrin-dependent endocytosis and micropinocytosis^10^. In addition, cholesterol in the plasma membrane plays an important role during entry^11^. In this regard, pseudotyped AcMNPV, which contains a glycoprotein from vesicular stomatitis virus (VSV-G), can transduce foreign genes into mammalian cells more efficiently, indicating that envelope proteins on the surface of AcMNPV are important for its entry into mammalian cells^12^. Upon modifying the AcMNPV envelope to include membrane-penetrating peptides, its entry into mammalian cells was enhanced^13^.

Apart from AcMNPV, no report have shown the use of BmNPV for gene transduction into mammalian cells. It is well-known that baculoviral entry to not only insect cells and also mammalian cells needs GP64. The amino acid sequence of GP64 from BmNPV (BmGP64) is slightly different from that of AcMNPV (AcGP64). Y153 in BmGP64, which corresponds to H155 in AcGP64, compromises function at a low pH and triggers membrane fusion of GP64 between the virus envelope and endosomal membranes^14^. The difference between the amino acid sequence of GP64 from BmNPV and that from AcMNPV causes non-permissivity of BmNPV in Sf-9 cells^14^. In addition, BomaNPV S2, which was isolated from the wild silkworm *Bombyx mandarina*, can replicate in Bm5, BmN and *Trichoplusia ni* cells (Tn-5B1-4 cells) and GP64 from BomaNPV S2 can enhance the infection of BmNPV in Tn-5B1-4 cells^15^, indicating that GP64 plays a crucial role in baculovirus host-range determination.

In this study, we tested BmNPV for gene transduction in mammalian cells. AcGP64-displaying BmNPV encoding the EGFP gene under the control of the cytomegalovirus (CMV) promoter was prepared from silkworm larvae. The AcGP64-displaying BmNPVs was transduced into human embryonic kidney 293T cells (HEK293T cells) and could successfully express EGFP protein.

## Results

### Transduction of recombinant BmNPV into HEK293T cells

**Figure 1:**
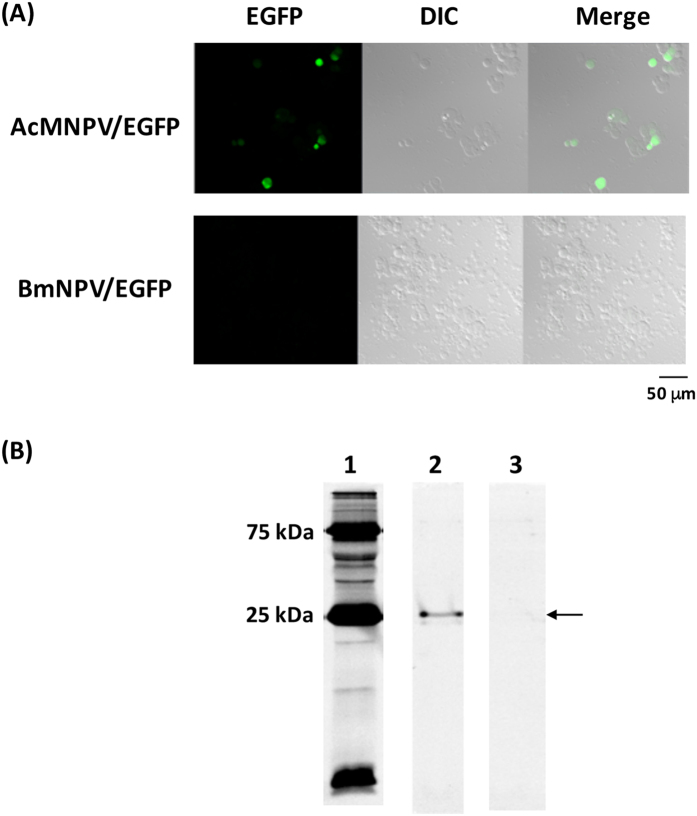
Transduction of AcMNPV/EGFP and BmNPV/EGFP into HEK293T cells. (**A**) Fluorescence microscopy of HEK293T cells transduced with each recombinant baculovirus. Each baculovirus was transduced into mammalian cells at M.O.I. 300, followed by cultivation for 48 h. After 48 h cultivation, trypsinized cells were put onto a glass slide and green fluorescence in the cells was observed using confocal laser scanning microscopy. (**B**) SDS-PAGE of baculovirus-transduced HEK293T cell homogenates. Lane 1: Marker, Lane 2: AcMNPV/EGFP, Lane 3: BmNPV/EGFP. Precision plus protein dual color standard (Bio-Rad) was used as a protein marker. An arrow indicates expressed EGFP.

We first investigated whether BmNPV could express a foreign gene in HEK293T cells. Recombinant AcMNPV/EGFP and BmNPV/EGFP (Table 1), both of which contain the EGFP gene under the control of the CMV promoter, were constructed and transduced into mammalian cells at a multiplicity of infection (M.O.I.) of 300. Green fluorescence was observed in mammalian cells transduced with AcMNPV/EGFP (Fig. 1A), and a band corresponding to EGFP was detected on an SDS-PAGE gel (Fig. 1B). However, with BmNPV/EGFP, no fluorescence or band on the SDS-PAGE gel were observed. This result indicates that BmNPV cannot transduce genes into HEK293T cells.

### Construction of an AcGP64-displaying BmNPV

We hypothesized that the difference in biochemical properties between AcGP64 and BmGP64 might be the cause of BmNPV’s inability to transduce a foreign gene into mammalian cells. Interestingly, AcGP64 can fuse with the plasma membrane in insect cells even at a relatively high pH, but the same is not true for BmGP64^14^. Therefore, a recombinant BmNPV displaying AcGP64 on its surface may be beneficial for the transduction of a foreign gene into mammalian cells using BmNPV.

**Figure 2:**
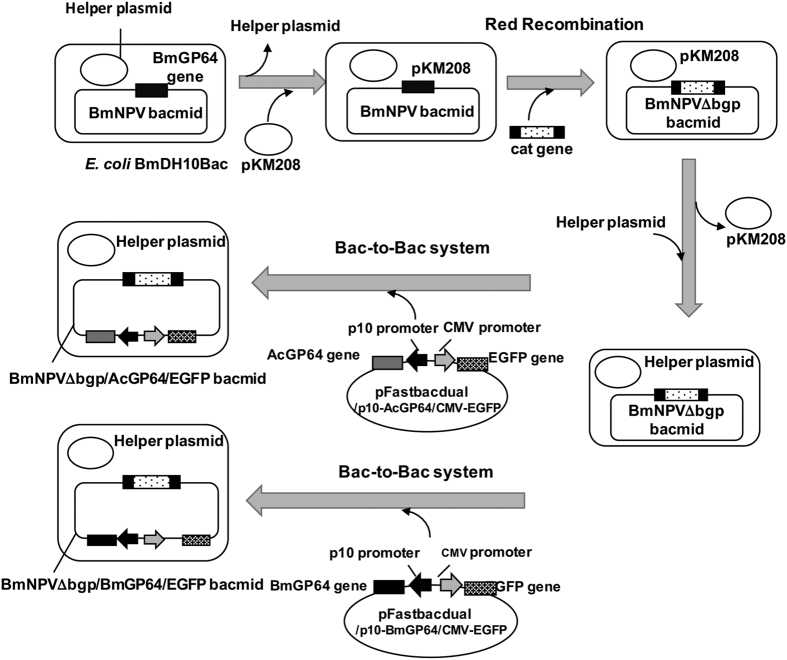
Scheme for each recombinant BmNPV bacmid. The BmGP64 gene was disrupted by a cat cassette using the Red recombination system to construct the ΔBmNPV bacmid. BmNPVΔbgp/AcGP64/EGFP and BmNPVΔbgp/BmGP64/EGFP bacmids were constructed using the Bac-to-Bac system and pFastbacdual/p10-AcGP64/CMV-EGFP and pFastbacdual/p10-BmGP64/CMV-EGFP, respectively, as shown.

**Figure 3:**
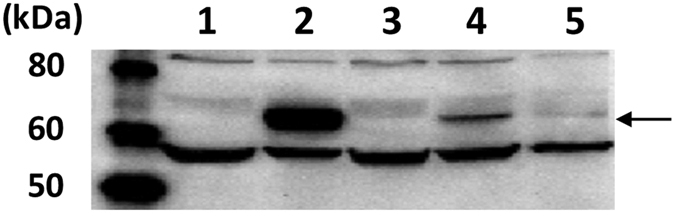
GP64 expression in insect cells. Western blot of GP64 using the homogenates of Bm5 cells transfected with each BmNPV bacmid. Rabbit anti-BmNPV GP64 antibody was used as a primary antibody. Lane 1: Mock, Lane 2: BmNPV bacmid, Lane 3: BmNPVΔbgp bacmid, Lane 4: BmNPVΔbgp/AcGP64/EGFP, Lane 5: BmNPVΔbgp/BmGP64/EGFP bacmid. Arrows indicate expressed AcGP64 or BmGP64.

Construction of each recombinant BmNPV bacmid is described in Fig. 2. First, the BmGP64 gene in the BmNPV bacmid was disrupted with the Red recombination system using pKM208. The BmGP64 gene-disrupted BmNPV (BmNPVΔbgp) bacmid was constructed and transfected into Bm5 cells to confirm the BmGP64 gene disruption. In baculoviruses, the deficiency of GP64 severely hinders its amplification^16^. As shown in Fig. 3, BmGP64 protein expression in Bm5 cells transfected with the BmNPVΔbgp bacmid was not detected but was present in the same cells transfected with the original BmNPV bacmid. In addition, the amplification of BmNPVΔbgp particles was not also observed. This results verifies that the BmGP64 gene in the BmNPV bacmid was disrupted.

Next, a BmNPVΔbgp bacmid containing the AcGP64 gene under the control of the p10 promoter and the EGFP gene under the control of the CMV promoter was constructed (Fig. 2) and designated as BmNPVΔbgp/AcGP64/EGFP (Table 1). As a negative control, we constructed a BmNPVΔbgp bacmid containing the BmGP64 gene under the control of the p10 promoter and the EGFP gene under the control of the CMV promoter (BmNPVΔbgp/BmGP64/EGFP bacmid, Fig. 2 and Table 1). BmNPVΔbgp/BmGP64/EGFP bacmid contains the same genomic DNA as BmNPVΔbgp/AcGP64/EGFP bacmid, except for the BmGP64 gene. These recombinant BmNPV bacmids were transfected into Bm5 cells to check for the expression of each GP64 gene. AcGP64 and BmGP64 were detected in Bm5 cells transfected with BmNPVΔbgp/AcGP64/EGFP and BmNPVΔbgp/BmGP64/EGFP bacmids, respectively (Fig. 3). However, GP64 expression under the control of p10 promoter was lower than that under the control of BmGP64 promoter in these cells.

**Figure 4:**
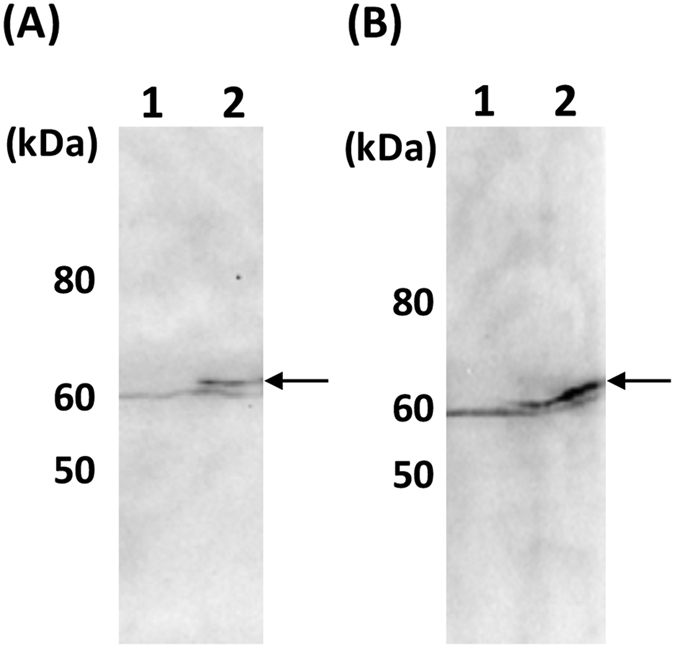
GP64 expression in silkworms. (**A**) Western blot of AcGP64 using the hemolymph of silkworm larvae injected with the BmNPVΔbgp/AcGP64/EGFP bacmid. Lane 1: Mock, Lane 2: BmNPVΔbgp/AcGP64/EGFP bacmid. (**B**) Western blot of BmGP64 using the hemolymph of silkworm larvae injected with the BmNPVΔbgp/BmGP64/EGFP bacmid. Lane 1: Mock, Lane 2: BmNPVΔbgp/BmGP64/EGFP bacmid. Arrows indicate expressed AcGP64 or BmGP64.

Constructed recombinant BmNPV bacmid DNAs were injected into silkworm larvae, respectively, and hemolymph was collected from silkworms. GP64 expression in hemolymph was confirmed by western blot. GP64 expression was detected in hemolymph from silkworm larvae injected with BmNPVΔbgp/AcGP64/EGFP or BmNPVΔbgp/BmGP64/EGFP bacmid (Fig. 4A,B). Titers of BmNPVΔbgp/AcGP64/EGFP and BmNPVΔbgp/BmGP64/EGFP both reached 1.1 × 10^9^ plaque-forming units (pfu)/ml in hemolymph at 7 days post injection of recombinant BmNPV bacmids. These results indicate that BmNPVΔbgp can replicate in silkworms when expressing either AcGP64 or BmGP64.

### Baculovirus transduction in mammalian cells

**Figure 5:**
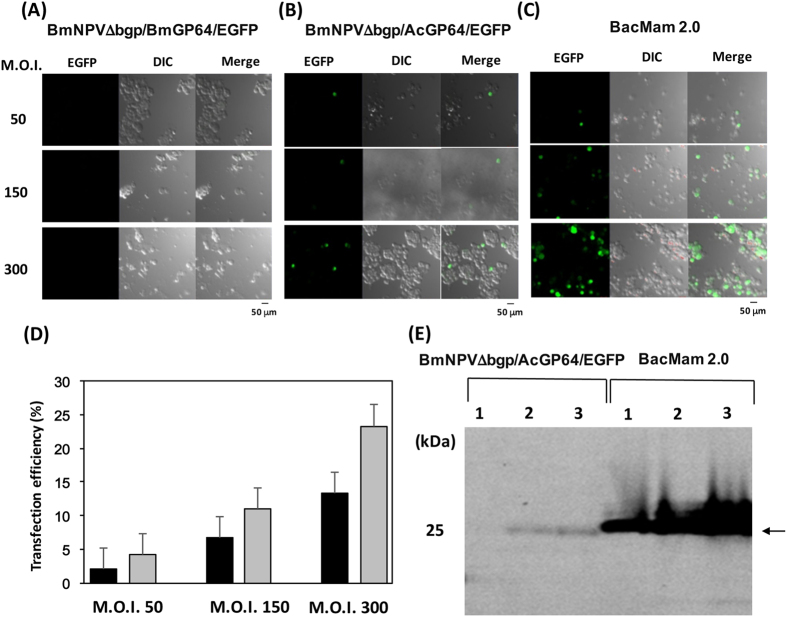
Transduction of each recombinant baculovirus into HEK293T cells at an M.O.I. of 50, 150 or 300. Fluorescence microscopy of HEK293T cells transduced with BmNPVΔbgp/BmGP64/EGFP (**A**), BmNPVΔbgp/AcGP64/EGFP (**B**), or the BacMam 2.0 (**C**). (**D**) Transduction efficiency of each recombinant baculovirus into HEK293T cells. Black and grey bars indicate BmNPVΔbgp/AcGP64/EGFP and the BacMam 2.0, respectively. (**E**) SDS-PAGE of baculovirus-transduced HEK293T cell homogenates. Lane 1: M.O.I. **=** 50, Lane 2: M.O.I. **=** 150, Lane 3: M.O.I. **=** 300. Arrow indicates expressed EGFP or EmGFP.

BmNPVΔbgp/AcGP64/EGFP and BmNPVΔbgp/BmGP64/EGFP were partially purified from hemolymph by sucrose density gradient centrifugation. As a positive control, the BacMam GFP transduction control (BacMam 2.0, Thermo Fisher Scientific) amplified in Sf-9 cells was used. BacMam 2.0 expressed emerald GFP (EmGFP) under the control of enhanced CMV promoter with Woodchuck hepatitis virus post-transcriptional regulatory element (WPRE). In addition, vesicular stomatitis virus glycoprotein (VSV-G) is expressed with AcGP64 on the envelope of BacMam 2.0 (Table 1). Each baculovirus was transduced into HEK293T cells at an M.O.I. of 50, 150 or 300. There was no green fluorescence observed in HEK293T cells transduced with BmNPVΔbgp/BmGP64/EGFP (Fig. 5A) or BmNPV/EGFP (Fig. 1), even at an M.O.I. of 300. However, green fluorescence was observed in some cells transduced with BmNPVΔbgp/AcGP64/EGFP (Fig. 5B and Fig. S1). Under the same conditions, stronger green fluorescence was observed in cells transduced with the BacMam 2.0 because EmGFP was expressed under the control of the stronger promoter, compared to that in BmNPVΔbgp/AcGP64/EGFP (Fig. 5C). The transduction efficiencies in cells transduced with the BacMam 2.0, BmNPVΔbgp/AcGP64/EGFP and BmNPVΔbgp/BmGP64/EGFP (M.O.I. of 300), which were calculated by counting green fluorescent cells, were 23, 13 and 0%, respectively (Fig. 5D). The expression of EGFP and EmGFP was also confirmed by SDS-PAGE (Fig. 5E). These results indicate that BmNPVΔbgp/AcGP64/EGFP can transduce the EGFP gene into HEK293T cells and that the replacement of BmGP64 with AcGP64 allows BmNPV to enter mammalian cells and express a foreign gene.

### GP64 expression level on the surface of each baculovirus

**Figure 6:**
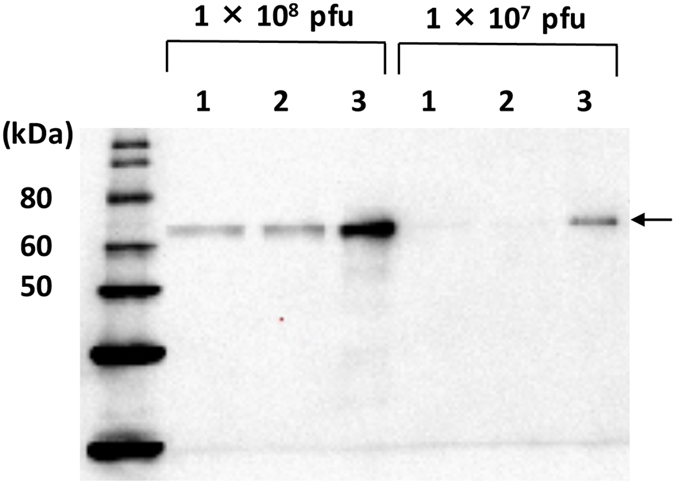
Western blot of GP64 from each baculovirus. Each virus was propagated on Bm5 (BmNPVΔbgp/AcGP64/EGFP and BmNPVΔbgp/BmGP64-EGFP) or Sf-9 (BacMam 2.0) cells and partially purified. Subsequently, 1 × 10^8^ or 1 × 10^7^ PFU of each virus was separated by SDS-PAGE, transferred to a PVDF membrane, and subjected to western blot analysis using rabbit anti-BmNPV GP64 polyclonal antibody. Lane 1: BmNPVΔbgp/AcGP64/EGFP, Lane 2: BmNPVΔbgp/BmGP64-EGFP, Lane 3: BacMam 2.0. Arrows indicate expressed AcGP64 or BmGP64.

The transduction efficiency of BmNPVΔbgp/AcGP64/EGFP was lower than that of the BacMam 2.0. We hypothesized that the amount of GP64 on the surface of the virus influences transduction efficiency because the GP64 expression level in Bm5 cells transfected with BmNPVΔbgp/AcGP64/EGFP bacmid was lower than that in Bm5 cells transfected with the BmNPV bacmid. BmNPVΔbgp/AcGP64/EGFP and BmNPVΔbgp/BmGP64/EGFP from silkworm larval hemolymph and the BacMam 2.0 from Sf-9 cultures were purified by sucrose density gradient centrifugation. Bands corresponding to GP64 were detected for all three baculoviruses (Fig. 6). However, the AcGP64 amount of BacMam 2.0 was approximately 10-fold higher than that of BmNPVΔbgp/AcGP64/EGFP or BmNPVΔbgp/BmGP64/EGFP. This result suggests that the GP64 expression level on the surface of BmNPV is crucial for baculovirus transduction into mammalian cells.

## Discussion

In this study, we found that BmNPV can transduce mammalian cells after the substitution of BmGP64 with AcGP64. This report is the first showing that BmNPV can transduce foreign genes into mammalian cells as well as AcMNPV. Transduction of BmNPV into mammalian cells is very advantageous because BmNPV can be prepared in the hemolymph of silkworm larvae at 10^9^ pfu/ml without any special equipment, even though specific diet is needed for silkworm rearing. However, the transduction efficiency of recombinant BmNPVΔbgp/AcGP64/EGFP constructed in this study was lower than that of the BacMam 2.0, which is based on AcMNPV. To use BmNPV for gene transduction into mammalian cells, its transduction efficiency needs to be improved. The low expression level of AcGP64 from BmNPVΔbgp/AcGP64/EGFP may be caused by the use of p10 promoter to express AcGP64 instead of native GP64 promoter. Normally, GP64 is expressed from its own promoter in cells infected by a baculovirus. AcGP64 is expressed from its own promoter in the BacMam 2.0 and from the p10 promoter in BmNPVΔbgp/AcGP64/EGFP. The p10 and polyhedrin promoters work at a very late stage of infection, but the GP64 promoter is an immediate early promoter, meaning that GP64 expression levels peak from 8 to 24 h after baculovirus infection^17^,^18^,^19^. GP64 is needed for baculovirus budding from infected cells^16^ and, therefore, it should be expressed before budding. Thus, GP64 expression from the p10 promoter may be achieved at a very late stage of infection, leading to inefficient GP64 expression in cells and inefficient display of GP64 on the baculovirus particles. To improve the transduction efficiency of BmNPV into mammalian cells, AcGP64 should be expressed on the surface of BmNPV from its own GP64 promoter.

In this study, we showed that BmNPV could transduce the EGFP gene into HEK293T cells after the substitution of BmGP64 with AcGP64. In a previous study, GP64 from BomaNPV S2, which was isolated from wild silkworms and can infect the cells of *B. mori*, *Spodoptera frugiperda* and *Trichoplusia ni*, allowed the enhancement of BmNPV infection of *T. ni* cells^15^. In addition, AcGP64 can fuse with the plasma membrane at a lower pH than BmGP64^14^. This finding indicates that AcGP64 facilitates its fusion with the plasma membrane at a higher pH compared to BmGP64. These results suggest that the difference in the fusing capacity of between AcGP64 and BmGP64 with the plasma membrane at a relatively high pH is one of the reasons why BmNPV cannot transduce foreign genes into mammalian cells.

## Materials and Methods

### Cell lines, viruses, and silkworms

Sf-9 cells were purchased from Thermo Fisher Scientific K.K. (Yokohama, Japan) and Bm5 cells were gifted by Prof. K. S. Boo (Insect Pathology Laboratory, School of Agricultural Biotechnology, Seoul National University, Seoul, Korea). Sf-9 and Bm5 cells were maintained at 27 °C in Sf-900II Serum-Free Medium (SFM; Thermo Fisher Scientific K.K.) supplemented with 1% fetal bovine serum (Thermo Fisher Scientific K.K.) and Antibiotic-Antimycotic (Thermo Fisher Scientific K.K.). HEK293T cells were purchased from American Type Culture Collection (Manassas, VA, USA) and maintained at 27 °C in Dulbecco’s Modified Eagle’s Medium (DMEM; Thermo Fisher Scientific K.K.) supplemented with 5% horse serum (Thermo Fisher Scientific K.K.) and 1% non-essential amino acids (Thermo Fisher Scientific K.K.) in the presence of 5% CO_2_. The BacMam GFP transduction control (BacMam 2.0, Thermo Fisher Scientific K.K) coding emerald GFP (EmGFP), was amplified in Sf-9 cells. Recombinant BmNPV bacmids constructed in this study were transfected into Bm5 cells in the presence of Cellfectin II (Thermo Fisher Scientific K.K.). Fourth instar silkworm larvae were purchased from Ehimesansyu (Nishiuwagun, Ehime, Japan) and were reared using Silkmate 2S (Nosan, Yokohama, Japan) as an artificial diet. To amplify recombinant BmNPVs in silkworm larvae, each recombinant BmNPV bacmid was mixed with DMRIE-C transfection reagent (Thermo Fisher Scientific K.K.) and injected into fifth instar larvae on first day, which were then reared for 4 to 7 days.

### Construction of each recombinant baculovirus

#### AcMNPV/EGFP

The CMV promoter sequence for AcMNPV/EGFP was PCR-amplified using CMV-F and CMV-R primers and pcDNA 3.1 (Thermo Fisher Scientific K.K.) as a template (Table 2). The polyhedrin promoter sequence in pFastbac1 (Thermo Fisher Scientific K.K.) was exchanged with the amplified CMV promoter sequence via an In-fusion reaction (Takara Bio Inc., Otsu, Japan). For this In-fusion reaction, pFastbac1 was PCR-amplified using pFastbac1-F and pFastbac1-R primers (Table 2). The constructed vector was designated as pFastbac/CMV. The EGFP gene amplified by PCR using EGFP-F and EGFP-R primers (Table 2) was inserted downstream of the CMV promoter in pFastbac/CMV. The constructed vector, pFastbac/CMV-EGFP, was transformed into *Escherichia coli* DH10Bac cells (Thermo Fisher Scientific K.K.), and the recombinant AcMNPV bacmid was extracted from a white transformant using an alkaline extraction method according to the Bac-to-Bac system (Thermo Fisher Scientific K.K.) protocol. The constructed recombinant AcMNPV bacmid was designated as AcMNPV/EGFP. This bacmid was transfected into Sf-9 cells, and its culture supernatant was collected as an AcMNPV/EGFP solution.

#### BmNPV/EGFP

pFastbac/CMV-EGFP was transformed into *E. coli* BmDH10Bac cells^4^, and the recombinant BmNPV bacmid was extracted from a white transformant using an alkaline extraction method according to the Bac-to-Bac system protocol. The constructed recombinant BmNPV bacmid was designated as BmNPV/EGFP. This bacmid was injected into silkworm larvae, and the collected hemolymph was used as the BmNPV/EGFP solution.

#### BmNPVΔbgp

*E. coli* BmDH10Bac cells containing the BmNPV bacmid and a helper plasmid containing the Tn7 transposase gene were used for this construction. First, the helper plasmid was removed from *E. coli* BmDH10Bac cells by several passages of this strain in tetracycline-free medium. pKM208 (Addgene, Cambridge, MA, USA) was transformed into the *E. coli* strain. The chloramphenicol acetyltransferase (cat) gene containing 50 bp of sequence, including the 5′-noncoding region of BmGP64 and the 3′-noncoding region of BmGP64, was PCR-amplified by using cat-F and cat-R primers (Table 2). The amplified cat gene was transformed into the *E. coli* strain harboring the BmNPV bacmid and pKM208^20^ and was cultured in the presence of chloramphenicol. The transformant was cultured at 37 °C to remove pKM208, and the helper plasmid containing the Tn7 transposase gene was transformed into the *E. coli* strain as described above. This new *E. coli* strain was then designated as *E. coli* BmDH10Bac/ΔBmGP64, harboring the BmNPVΔbgp bacmid and the helper plasmid containing the Tn7 transposase gene. Disruption of the BmGP64 gene in the BmNPVΔbgp bacmid was confirmed by PCR using cat-F and cat-R primers.

#### BmNPVΔbgp/AcGP64/EGFP

The polyhedrin promoter sequence in pFastbacdual (Thermo Fisher Scientific K.K.) was exchanged with the amplified CMV promoter sequence via an In-fusion reaction. For this In-fusion reaction, pFastbacdual was PCR-amplified using pFstbac1-F and pFastbac1-R primers (Table 2). The constructed vector was designated as pFastbacdual/p10/CMV. The EGFP and AcGP64 genes were inserted downstream of the CMV and p10 promoters in pFastbacdual/p10/CMV, respectively. The AcGP64 gene was PCR-amplified using AcGP64-F and Ac-GP64-R primers (Table 2) and AcMNPV genomic DNA as a template. The constructed vector (pFastbacdual/p10-AcGP64/CMV-EGFP) was transformed into *E. coli* BmDH10Bac/ΔBmGP64, and the recombinant BmNPV bacmid was extracted from a white transformant. The constructed recombinant BmNPV bacmid was designated as the BmNPVΔbgp/AcGP64/EGFP bacmid. This bacmid was injected into silkworm larvae, and its hemolymph was used as a BmNPVΔbgp/AcGP64/EGFP solution.

#### BmNPVΔbgp/BmGP64/EGFP

The EGFP and BmGP64 genes were inserted downstream of the CMV and p10 promoters in pFastbacdual/p10/CMV, respectively. The BmGP64 gene was PCR-amplified using BmGP64-F and Bm-GP64-R primers (Table 2) and the BmNPV bacmid as a template. The constructed vector (pFastbacdual/p10-BmGP64/CMV-EGFP) was transformed into *E. coli* BmDH10Bac/ΔBmGP64 cells, and the recombinant BmNPV bacmid was extracted from a white transformant. The constructed recombinant BmNPV bacmid was designated as BmNPVΔbgp/BmGP64/EGFP. This bacmid was injected into silkworm larvae, and its hemolymph was used as a BmNPVΔbgp/BmGP64/EGFP solution.

Construction of all baculovirus bacmids in this study was confirmed by PCR using primers, M13-F and M13-R (Table 2).

### SDS-PAGE and western blot

Proteins were separated by sodium dodecyl sulfate-polyacrylamide gel electrophoresis (SDS-PAGE) using 10% or 12% acrylamide gels that were subsequently subjected to western blotting. After SDS-PAGE, proteins were blotted onto a polyvinylidene fluoride (PVDF) membrane using the Mini Trans-Blot Electrophoretic Transfer Cell (Bio-Rad, Hercules, CA, USA). After blocking in 5% skim milk with Tris-buffered saline containing 0.1% Tween 20 (TBST), the membrane was probed with 1:5000 rabbit anti-BmNPV GP64 polyclonal antibody (Biogate, Gifu, Japan). This antibody can recognize both AcGP64 and BmGP64. The membrane was washed with TBST and incubated for 1 h in 1:20,000 anti-mouse or anti-rabbit IgG antibody labeled with horseradish peroxidase (GE Healthcare, Buckinghamshire, UK). Detection was performed using ECL Plus Western blotting reagent (GE Healthcare Japan, Tokyo, Japan). Specific bands were detected on a Fluor-S MAX MultiImager (Bio-Rad). To detect GFP-specific green fluorescent protein bands, the samples were mixed with sample buffer^21^ without boiling and separated by SDS-PAGE. Green fluorescent bands were detected using a Molecular Imager FX (Bio-Rad).

### Partial purification of each recombinant baculovirus

AcMNPV/EGFP and BacMam 2.0 were collected by ultracentrifugation (122,000 × *g*, 1 h) from Sf-9 culture supernatants. Collected recombinant AcMNPVs were resuspended in a small volume of phosphate-buffered saline (PBS, pH 7.4) and partially purified by sucrose density gradient centrifugation (20–60%; 122,000 × *g*; 3 h). A white band was collected, and recombinant AcMNPVs were pelleted by ultracentrifugation (122,000 × *g*; 1 h). Collected recombinant AcMNPVs were resuspended in a small volume of PBS, and the virus suspension was dialyzed with PBS using a 300-kDa cutoff dialysis membrane (Spectrum Labs, Rancho Dominguez, CA, USA). Recombinant AcMNPVs were stored at 4 °C.

The hemolymph of the silkworm larvae was centrifuged at 122,000 × *g* for 1 h, and the pellet was resuspended in PBS using sonication. Recombinant BmNPVs were partially purified from this suspension by sucrose density gradient centrifugation. A white band was collected, and recombinant BmNPVs were pelleted by ultracentrifugation (122,000 × *g*; 1 h). Collected recombinant BmNPVs were resuspended in a small volume of PBS, and the virus suspension was dialyzed with PBS using a 300-kDa cutoff dialysis membrane (Spectrum Labs). Recombinant BmNPVs were stored at 4 °C.

### Determination of baculovirus titers

Titers of recombinant AcMNPV and BmNPV were determined using the methods described in previous reports^22^,^23^. In the case of recombinant AcMNPV, AcIE-F and AcIE-R primers (Table 2) were used, and in the case of recombinant BmNPV, BmIE-F and BmIE-R primers (Table 2) were used.

### Transduction of recombinant baculoviruses into mammalian cells

The three recombinant baculoviruses (BacMam 2.0, BmNPVΔbgp/AcGP64/EGFP and BmNPVΔbgp/BmGP64/EGFP) were transduced into 2 × 10^5^ HEK293T cells at an M.O.I. of 50, 150, or 300. After 48 h cultivation, trypsinized cells were put onto a glass slide and green fluorescence in the cells was observed using confocal laser scanning microscopy (LSM700, Carl Zeiss Japan, Tokyo, Japan). Transduction efficiencies were calculated by counting the green fluorescent cells in 5 different pictures in a single experiment.

## Additional Information

**How to cite this article**: Kato, T. *et al*. Gene transduction in mammalian cells using *Bombyx mori* nucleopolyhedrovirus assisted by glycoprotein 64 of *Autographa californica* multiple nucleopolyhedrovirus. *Sci. Rep*. **6**, 32283; doi: 10.1038/srep32283 (2016).

## Supplementary Material

Supplementary Information

## Figures and Tables

**Table 1:** Used recombinant baculoviruses.

Name	Details
AcMNPV/EGFP (Constructed)	AcMNPV encoding EGFP gene under the control of CMV promoter
BmNPV/EGFP (Constructed)	BmNPV encoding EGFP gene under the control of CMV promoter
BmNPVΔbgp (Constructed)	BmGP64 gene-disrupted BmNPV
BmNPVΔbgp/AcGP64/EGFP (Constructed)	BmNPVΔbgp encoding AcGP64 gene and EGFP gene under the control of p10 promoter and CMV promoter, respectively
BmNPVΔbgp/BmGP64/EGFP (Constructed)	BmNPVΔbgp encoding BmGP64 gene and EGFP gene under the control of p10 promoter and CMV promoter, respectively
BacMam 2.0 (Thermo Fisher Scientific)	AcMNPV encoding EmGFP gene under the control of enhanced CMV promoter with WPRE and displaying VSV-G and AcGP64 on the envelope.

**Table 2:** Primers used in this study.

Name	5′ → 3′
CMV-F	CCGGAATATTAATAGGTTGACATTGATTA
CMV-R	CTCAAGCAGTGATCACCATAGAGCCCAC
pFastbac1-F	TGATCACTGCTTGAGCCTA
pFastbac1-R	CTATTAATATTCCGGAGTA
EGFP-F	ATGAATTCATCATGGTGAGCAAGGGCGCCG
EGFP-R	TAGCGGCCGCCTACTTGTACAGCTCATCC
cat-F	TGCTACTAGTAAATCAGTCATACCAAGGCTTCGATAAGAAACACACAAGCCCATATGAATATCCTCCTTA
cat-R	ACAAATAATGATACAATTTTTATTATTACATTTAATATTGTCTACTATTATTGTGTAGGCTGGAGCTGCT
AcGP64-F	AGGCCCCGGGATGCTACTAGTAAATCAGTCAG
AcGP64-R	GGCGCCCGGGTTAATATTGTCTATTACGGTTTC
BmGP64-F	ACTTCCCGGGATGGTAGGCGCTATTGTTTTATAC
BmGP64-R	AGCGGCCCGGGTTAATATTGTCTACTATTACGG
AcIE-F	CCCGTAACGGACCTCGTACTT
AcIE-R	TTATCGAGATTTATTTGCATACAACAAG
BmIE-F	CCCGTAACGGACCTTGTGCTT
BmIE-R	TTATCGAGATTTATTTACATACAACAAG
M13-F	AGCGGATAACAATTTCACACAGG
M13-R	CCCAGTCACGACGTTGTAAAACG
