# Supplementary material for: Gene transduction in mammalian cells using Bombyx mori nucleopolyhedrovirus assisted by glycoprotein 64 of Autographa californica multiple nucleopolyhedrovirus
Source: Sci Rep. 2016 Aug 26;6:32283. doi: 10.1038/srep32283 (PMC4999795; doi:10.1038/srep32283)
Supplement: Supplementary Information [file srep32283-s1.pdf]

Gene transduction in mammalian cells using *Bombyx mori* nucleopolyhedrovirus assisted by glycoprotein 64 of *Autographa californica* multiple nucleopolyhedrovirus

Tatsuya Kato,[1,2,3*] Saki Sugioka,[2] Kohei Itagaki,[2] and Enoch Y, Park[1,2,3*]

[1] Laboratory of Biotechnology, Green Chemistry Research Division, Research Institute of Green Science and Technology, Shizuoka University, 836 Ohya Suruga-ku, Shizuoka, 422-8529, Japan

[2] Laboratory of Biotechnology, Department of Applied Biological Chemistry, Faculty of Agriculture, Shizuoka University, 836 Ohya, Suruga-ku, Shizuoka, 422-8529, Japan

[3] Laboratory of Biotechnology, Department of Bioscience, Graduate School of Science and Technology, Shizuoka University, 836 Ohya, Suruga-ku, Shizuoka, 422-8529, Japan

E-mail:
kato.tatsuya@shizuoka.ac.jp (TK)
sakicsxxx@yahoo.co.jp (SS)
pixy_007bc@yahoo.co.jp (KI)
park.enoch@shizuoka.ac.jp (EYP)

[*] Correspondence
Tatsuya Kato (kato.tatsuya@shizuoka.ac.jp); Enoch Y. Park (park.enoch@shizuoka.ac.jp)

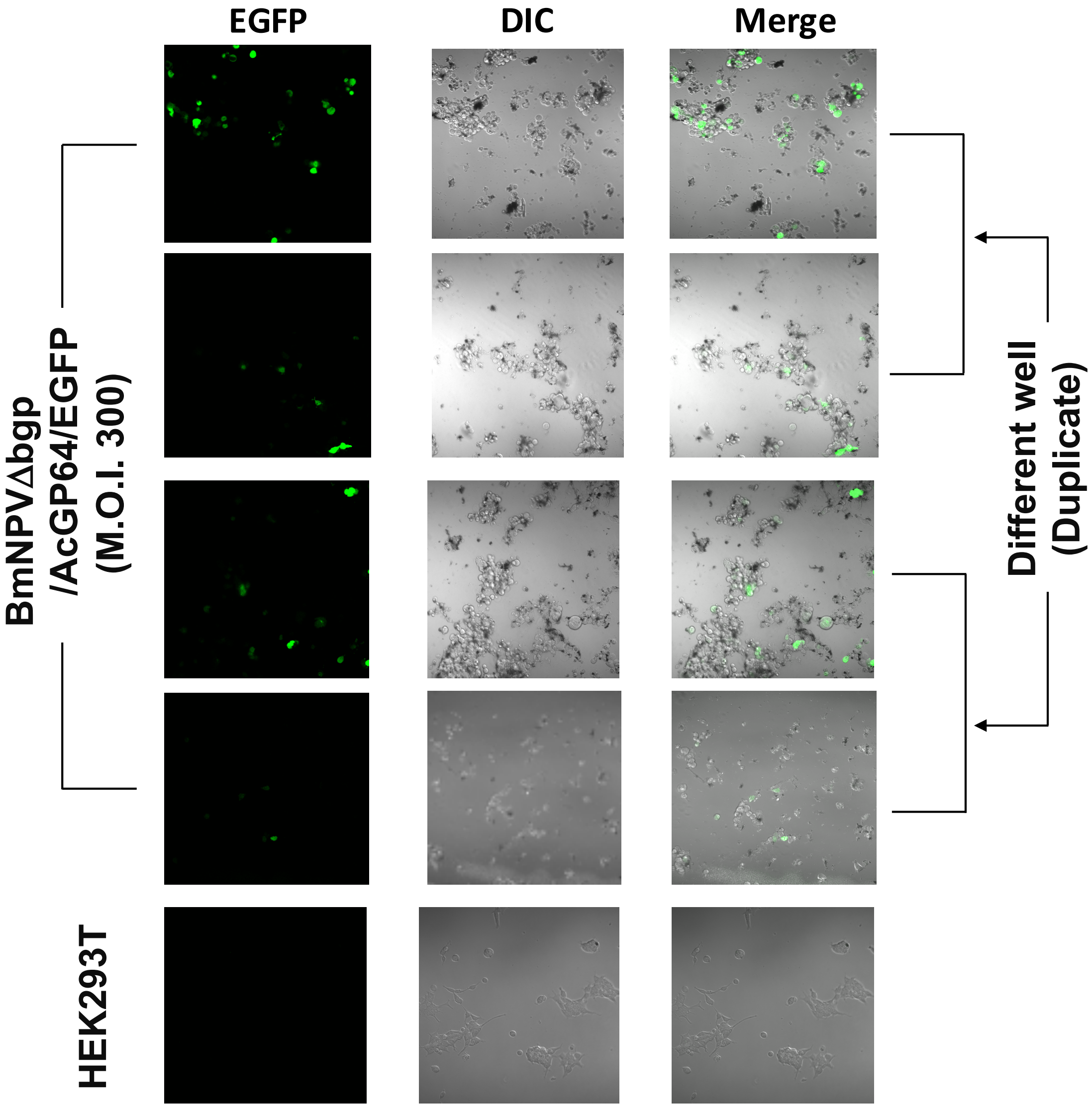

Figure S1. Fluorescence microscopy of HEK293T cells transduced with BmNPVΔbgp/AcGP64/EGFP. Transduction of recombinant BmNPVΔbgp/AcGP64/EGFPs into HEK293T cells at an M.O.I. of 300. Each transduction efficiency of recombinant BmNPVΔbgp/AcGP64/EGFPs into HEK293T cells was 7.8%, 9.5%, 12.7% and 8.3% judging by counting the number of fluorescent cells.
